# Cyclosporine-Induced Gingival Hyperplasia in a Patient With Lichen Planopilaris: Misfortunes Never Come Singly!

**DOI:** 10.7759/cureus.42531

**Published:** 2023-07-27

**Authors:** Vasileios Zisis, Dimitrios Andreadis, Rafaelia Karpouzi, Theodora Karadagli, Athanasios Poulopoulos

**Affiliations:** 1 Oral Medicine/Pathology, Aristotle University of Thessaloniki, Thessaloniki, GRC; 2 Prosthodontics, Aristotle University of Thessaloniki, Thessaloniki, GRC

**Keywords:** gingivectomy, lichen planopilaris, immunosuppression therapy, cyclosporine-a, gingival hypertrophy

## Abstract

Cyclosporine A constitutes an immunosuppressive medication administered against various autoimmune and autoinflammatory disorders as well as against graft versus host disease. Its most well-known oral adverse effect is gingival hyperplasia. The aim of this study is to report a persistent case of a patient with lichen planopilaris with alopecia treated with cyclosporine leading to the manifestation of gingival hypertrophy. A female patient aged 38 years old was referred to the Department of Oral Medicine/Pathology, Dental School, Aristotle University of Thessaloniki, Greece complaining about gum bleeding, halitosis, and a persistent gingival enlargement, which appeared two months ago. According to her medical history, lichen planopilaris was diagnosed six months ago and was initially treated for 40 days with methylprednisolone 16 mg twice per day without improvement, and was replaced by cyclosporine A 200 mg per day. The clinical oral examination revealed gingival enlargement at areas #34-43, 22-23, and 25-27 without any lesion of lichen planus. The level of oral hygiene was satisfactory, with a limited amount of tartar and plaque. Bleeding on probing was also noticed, and pseudopockets of 5 mm were observed. The serum levels of cyclosporine were 473,60 μg/L, with a normal range, regarding repercussions in the oral cavity, up to 200 μg/L. A decrease of cyclosporine dosage to 150 mg was performed. After 15 days, the clinical appearance significantly improved, and a biopsy was done. The microscopic findings showed mild ulceration and inflammatory infiltrates, together with the abundant presence of collagen stroma, without any sign of malignancy. According to the literature, the high dosage of cyclosporine, its relevant high serum levels, and the presence of plaque were responsible for the manifestation of gingival hypertrophy.

## Introduction

Many pharmaceutical agents are related to gingival overgrowth. Phenytoin, an antiepileptic drug that was first administered in 1937, was the first to be linked with gingival enlargement, and the first study studying it was published in 1939 [1]. Gingival enlargement is defined as the pathological hypertrophy and/or hyperplasia of gingival tissues as a result of excessive extracellular matrix expansion. Drug-induced gingival overgrowth (DIGO) pathology's underlying molecular mechanisms remain obscure and a wide variety of medications may induce DIGO. The medications most frequently linked to DIGO are phenytoin, cyclosporine, and nifedipine [2]. DIGO manifests on average in 71, 262, and 37 days, after administration of immunosuppressants, calcium channel blockers, and anticonvulsants, respectively [3]. According to cross-sectional studies, the prevalence of DIGO is estimated to be 30% for nifedipine, 30% for diltiazem, 20% for verapamil, 70% for phenytoin, and 50% to 80% for cyclosporine [4]. DIGO typically begins to develop within the first three months and reaches a plateau between nine and 12 months [5]. The immunosuppressive drug cyclosporine A (CsA), a calcineurin inhibitor, has revolutionized transplantology [6]. The decision to begin CsA therapy should be based on a risk-benefit analysis [6]. In cases of severe liver damage, severe and uncontrolled hypertension, malignant neoplasms, and concurrent use of psoralen plus ultraviolet A (PUVA), the use of CsA is not indicated under any circumstances [6]. The main oral manifestation of CsA is gingival enlargement and hypertrophy, which may lead to pseudo pockets, gingivitis, periodontitis, and tooth loss [7]. These clinical signs may be accompanied by tooth decay, pain, difficulty in eating, bleeding, and halitosis since the application of effective oral hygiene is obstructed by gingival enlargement [8]. As a result, the quality of life is diminished, since 53% of patients under CsA treatment post kidney transplantation and 8% to 85% of patients under CsA treatment for other disorders experience DIGO [6], which arises normally in the first three to six months post administration of treatment, usually affecting in the first place the interdental papillae, especially the labial side rather than the palatal or lingual side. Subsequently, the papillae start to enlarge and the entire tooth crown may be covered [9]. Lack of oral hygiene is associated with more severe hypertrophy [10], which is supported by the fact that salivary pro-inflammatory cytokines like interleukin (IL)-1, IL-6, and IL-8 play a role in the pathogenesis of CsA-induced gingival hyperplasia, and vice versa, maintaining proper periodontal health alleviates the severity of the hypertrophy [11]. The CsA-associated gingival hypertrophy has a male predilection, preferring older patients [7]. Apart from oral hygiene, sex, and age of the patients, other key factors include the length of the treatment and the dosage of the medication administered [6]. Interestingly, other calcineurin inhibitors (i.e., tacrolimus) have no or minor effect on gingival enlargement. The concurrent administration of calcium channel blockers enhances dramatically the possibility of DIGO (from 25% to 51.9%) [12]. The differential diagnosis of DIGO includes von Recklinghausen disease, hereditary gingival fibromatosis, neoplasia, and metastases [6-13]. The aim of this study is to present an interesting case of a 38-year-old female patient who received cyclosporine due to persistent lichen planopilaris and eventually manifested gingival enlargement.

## Case presentation

**Figure 1:**
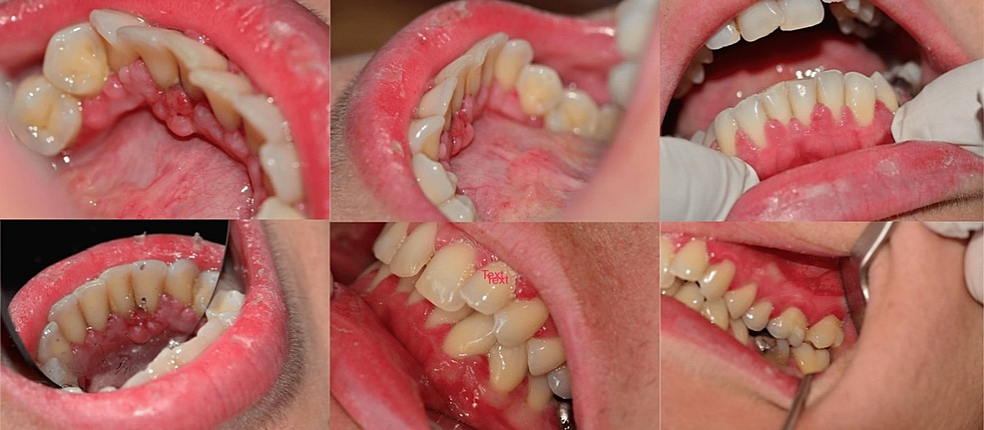
Clinical images of the areas manifesting gingival enlargement.

A 38-year-old female patient was referred to the Department of Oral Medicine/Pathology, Dental School, Aristotle University of Thessaloniki, Greece complaining about gum bleeding, halitosis, and a persistent gingival enlargement, which first appeared two months ago (Figure 1).

Before the examination, the patient provided written informed consent. This form was approved by the School of Dentistry, Aristotle University of Thessaloniki, Greece, and was in accordance with Helsinki Declaration for research and patient ethics. A thorough examination followed. Her medical history revealed a diagnosis of lichen planopilaris six months ago in the scalp, leading to extensive hair loss and alopecia. The lichen proved to be persistent since it did not respond to the 40-day therapeutic regimen of methylprednisolone 16 mg twice per day. Subsequently, cyclosporine A 200 mg per day was prescribed. The lichen planopilaris responded to the treatment, but after the cyclosporine administration, the interdental papillae began to manifest hypertrophy and continued to grow. The clinical examination of the oral cavity showed no signs of (oral) lichen planus but an extensive gingival enlargement at the areas #34-43, 22-23, and 25-27. The level of oral hygiene was satisfactory, with a limited amount of tartar and plaque. Bleeding on probing was also noticed, and pseudo pockets of 5 mm were observed. The serum level of cyclosporine was 473,60 μg/L on the blood examination when the normal level is up to 200 μg/L (normal level with regard to the appearance of gingival hypertrophy). The daily dose of cyclosporine was adjusted to 150 mg daily. The clinical appearance significantly improved, and a biopsy was done after 15 days. The microscopic findings included mild ulcerations and inflammatory infiltrates, together with a wide presence of collagenous subepithelial stroma, without any sign of malignancy.

## Discussion

The patient presented with a form of lichen planus, which belonged to the minority of cases necessitating extensive immunosuppression. Cyclosporine succeeded in controlling the disease; however, provoked the adverse effect of gingival enlargement. The key factor is the patient’s serum level of cyclosporine at 473,60 μg/L because, according to the literature, serum levels of less than 200 μg/L do not affect the gingival fibroblasts, whereas serum levels ranging from 400 to 800 μg/L affect the fibroblasts’ viability and proliferation [4]. The underlying pathogenetic mechanism involves mediators, such as angiotensin II, endothelin-1, fibrogenic cytokines, transforming growth factor, connective tissue growth factor, insulin-like growth factor, platelet-derived growth factor, mast cell chymase, and tryptase enzymes, which induce the extensive fibroblast differentiation [14]. Each drug induces fibrosis on a different level; cyclosporine provokes less fibrosis, compared to nifedipine, which provokes intermediate fibrosis, and phenytoin, which provokes the most fibrosis [14]. The connective tissue growth factor and the transforming growth factor play a more important role in gingival hypertrophy due to phenytoin intake whereas the keratinocyte growth factor (KGF) plays a more important role in gingival hypertrophy due to cyclosporine intake. In phenytoin-induced gingival hyperplasia, KGF is more important in CsA-induced gingival hyperplasia [15]. Finally, an imbalance between the phenomena of cell renewal, cell proliferation, and programmed cell death (apoptosis) may also induce gingival hypertrophy [9]. In the long term, controlling dental plaque is the main focus of treatment. Regular tooth brushing, tartar removal techniques (scaling, sandblasting), interdental cleaning with dental floss or an irrigator, and mouthwash use are all preventive methods in daily oral hygiene [10]. Then, the possibility should be taken into consideration that the disease-inducing medication be substituted [7]. Due to the limited number of available treatments, CsA substitution is quite challenging. Tacrolimus can be used in its place since patients treated with CsA have a significantly higher risk of developing gingival hyperplasia than patients treated with tacrolimus [11]. It has been demonstrated that using azithromycin (azithromycin influences the function of fibroblasts [16]) in conjunction with CsA lessened the severity of DIGO [6], whereas rinsing with chlorhexidine lessens the severity of gingival enlargement. It is important to wait six to 12 months after any drug substitution attempt to deduce whether the gingival enlargement has been resolved, and in case of esthetic issues or when the oral cavity's functionality is compromised, gingivectomy is applied [6-17]. Gingivectomy may be performed with periodontal knives, scalpel, or laser, with the aim of eradicating pseudo pockets and rehabilitation of the normal gingival architecture [6]. Up to 34% of the cases recur within 18 months of periodontal surgery [8]. Laser gingivectomy is correlated to better results and less recurrences [8]. In the case of the study, the patient refused any surgical intervention out of fear of post-surgical complications such as edema and pain. She was further referred to her dentist for teeth cleaning.

## Conclusions

The use of CsA can cause gingival overgrowth, which can also be brought on by other elements like plaque or bacteria. Therefore, it is crucial to educate patients about the importance of maintaining good oral hygiene and the requirement to see a dentist every six months. Surgical procedures must be used when gingival hyperplasia significantly impairs either the esthetics or the function of the oral cavity. Blood pressure should be monitored, lab tests should be run, and the patient's oral cavity should receive special attention while receiving CsA therapy. It is crucial to take an interdisciplinary approach. A dentist, doctor, or hygienist's assistance is crucial in the early detection of gingival hyperplasia brought on by the use of CsA and is required in the treatment of this condition.
